# Pluripotent Stem Cell-Based Approaches to Explore and Treat Optic Neuropathies

**DOI:** 10.3389/fnins.2018.00651

**Published:** 2018-09-20

**Authors:** Oriane Rabesandratana, Olivier Goureau, Gaël Orieux

**Affiliations:** Sorbonne Université, INSERM, CNRS, Institut de la Vision, Paris, France

**Keywords:** glaucoma, retinal ganglion cells (RGCs), human iPSCs, cell transplantation, disease modeling

## Abstract

Sight is a major sense for human and visual impairment profoundly affects quality of life, especially retinal degenerative diseases which are the leading cause of irreversible blindness worldwide. As for other neurodegenerative disorders, almost all retinal dystrophies are characterized by the specific loss of one or two cell types, such as retinal ganglion cells, photoreceptor cells, or retinal pigmented epithelial cells. This feature is a critical point when dealing with cell replacement strategies considering that the preservation of other cell types and retinal circuitry is a prerequisite. Retinal ganglion cells are particularly vulnerable to degenerative process and glaucoma, the most common optic neuropathy, is a frequent retinal dystrophy. Cell replacement has been proposed as a potential approach to take on the challenge of visual restoration, but its application to optic neuropathies is particularly challenging. Many obstacles need to be overcome before any clinical application. Beyond their survival and differentiation, engrafted cells have to reconnect with both upstream synaptic retinal cell partners and specific targets in the brain. To date, reconnection of retinal ganglion cells with distal central targets appears unrealistic since central nervous system is refractory to regenerative processes. Significant progress on the understanding of molecular mechanisms that prevent central nervous system regeneration offer hope to overcome this obstacle in the future. At the same time, emergence of reprogramming of human somatic cells into pluripotent stem cells has facilitated both the generation of new source of cells with therapeutic potential and the development of innovative methods for the generation of transplantable cells. In this review, we discuss the feasibility of stem cell-based strategies applied to retinal ganglion cells and optic nerve impairment. We present the different strategies for the generation, characterization and the delivery of transplantable retinal ganglion cells derived from pluripotent stem cells. The relevance of pluripotent stem cell-derived retinal organoid and retinal ganglion cells for disease modeling or drug screening will be also introduced in the context of optic neuropathies.

## Introduction

Sight is defined first as the faculty to detect light (non-image forming visual functions), then enabling to form an image of the environment (image forming visual function). This sense appeared very early in the evolution (Gehring, 2002) showing that this faculty is essential for many species to apprehend their environment and survive. Vision impairment is particularly disabling, especially irreversible and untreatable blindness that is often due to degeneration of the retina, the light-sensitive tissue located at the back of the eye. Retina consists of a stratified neural layer and the non-neural supporting retinal pigmented epithelium (RPE). Virtually all retinal dystrophies can be separated in two major groups; on one hand, those affecting photoreceptor and RPE cells including inherited retinal dystrophies (i.e., Retinitis Pigmentosa) and Age-related Macular Degeneration. On the other hand, retinal ganglion cell (RGC) disorders affecting the output neurons of the retina that project through the optic nerve to all retinal targets in the brain. Since the optic nerve consists of RGC axons, RGC disorders and optic neuropathies are usually grouped together. RGCs disorders are common, highlighting the vulnerability of these cells. Some optic neuropathies are very common with glaucoma which is the first cause of irreversible blindness while others are scarce like Leber’s hereditary optic neuropathy (LHON). There is currently no treatment available for inherited optic neuropathies such as LHON or dominant optic atrophy (DOA), and for glaucoma, current treatments aim to lower the intraocular pressure (IOP). However, RGC death can progress despite lowered IOP and no current treatments promoting RGC surveillance and regeneration are available (Almasieh et al., 2012; Sluch and Zack, 2014; Greco et al., 2016; Jonas et al., 2017). Moreover, RGC degeneration is often silent with no conscious impact on vision acuity before reaching a high percent of cell loss. Since RGC disorders can be detected at an advanced stage of the disease, innovative treatments for patients showing advanced RGC degeneration is required. The retina presents some attractive features for innovative treatments dedicated to neurodegenerative diseases, including gene therapy, cell therapy or prosthetic therapy. It is a more accessible structure, compared to other structures of the central nervous system and as part of the eye, the retina is relatively isolated from the rest of the body insuring limited systemic diffusion of the therapeutic product. Finally, structural and functional benefits or adverse effects can be easily followed-up by imaging, electrophysiology and behavioral tests. Cell-based therapies have been largely explored over the past few decades, notably for retinopathies due to photoreceptor and/or RPE cell death (Goureau et al., 2014; Jayakody et al., 2015; Zarbin, 2016; Aghaizu et al., 2017; Jones et al., 2017; Llonch et al., 2018), and more recently for RGC disorders (Sluch and Zack, 2014; Chamling et al., 2016; Daliri et al., 2017).

Cell therapy can address two major issues. One objective is to deliver a trophic and neuroprotective support (Mead et al., 2015; Ding et al., 2017; Park et al., 2017) in order to limit or to stop the degenerative process and the worsening of the visual deficit. This strategy should be compared to pharmacological approaches designed to deliver neuroprotective agents. The other one is more ambitious as transplanted cells may replace lost cells and contribute to functional restoration. Regenerative medicine has experienced a huge expansion for the past two decades, since the isolation of human embryonic stem cells (ESCs) (Thomson et al., 1998). Indeed, human ESCs can be maintained virtually endlessly in undifferentiated state *in vitro* and can differentiate into all the three germ layers (endoderm, mesoderm, and ectoderm). In 2006, the group of S. Yamanaka generated another type of pluripotent stem cells (PSCs) by reprogramming mouse fibroblasts with four specific transcription factors, POU domain, class 5 transcription factor 1 (Pou5f1, also known as Oct3/4), SRY (sex determining region Y)-box 2 (Sox2), myc proto-oncogene protein (c-Myc) and Kruppel-like factor 4 (Klf4) (Takahashi and Yamanaka, 2006). Shortly after, this group validated the reprogramming of human cells with the same four human-homologous factors (Takahashi et al., 2007). At the same time, the group of J. A. Thomson obtained similar results with a slightly different combination of reprogramming factors comprising OCT4, SOX2, Nanog homeobox (NANOG) and Lin-28 homolog A (LIN28) (Yu et al., 2007). These cells, named induced pluripotent stem cells (iPSCs) display almost all the ESC features and represent an incredibly promising source of cells for transplantation approaches. Additionally, human iPSCs, overcome ethical issues inherent to the use of human embryonic material. Following its original discovery, different methods of delivery of reprogramming factors have been designed, notably to avoid integrative approaches that would represent an obstacle to clinical application (Junying et al., 2009; González et al., 2011).

One key point for cell therapy is to obtain a well-characterized cell population with the appropriate identity at a specific stage of differentiation. This requires recapitulating *in vivo* development, in a stepwise fashion of specification. The generation of retinal cells involves the generation of anterior neuroblasts, then the commitment into eye field lineage, and afterwards, the specification into neural retina or RPE identity (Graw, 2010; Jayakody et al., 2015; Stenkamp, 2015; Rathod et al., 2018). During the last decade, most efforts have been concentrated, successfully, on the generation of photoreceptors and RPE cells (Lamba et al., 2006; Osakada et al., 2008; Meyer et al., 2009; Nakano et al., 2012; Reichman et al., 2014, 2017; Zhong et al., 2014). Several human clinical trials have been approved and already started for RPE cell replacement (Schwartz et al., 2016; Zarbin, 2016; Mandai et al., 2017; da Cruz et al., 2018; Kashani et al., 2018).

The literature dedicated to the generation of PSC-derived RGCs and to cell therapy designed to RGC disorders is less abundant. One explanation may be the challenging goal of optic nerve regeneration that may look daunting to some. However, important progress has been achieved in order to generate well characterized transplantable cells (Gill et al., 2014; Tanaka et al., 2016; Teotia et al., 2016; Liu et al., 2017; Sluch et al., 2017; Langer et al., 2018) and to address the question of axonal regeneration (Park et al., 2008; Sun et al., 2011; de Lima et al., 2012; Benowitz et al., 2017; Calkins et al., 2017; Laha et al., 2017). In this review, we discuss the feasibility of regenerative strategies applied to RGC disorders such as glaucoma and inherited optic neuropathies using PSCs. For this purpose, the different strategies for the generation of PSC-derived RGCs are described. Complementary cell therapy approaches dedicated to deliver a trophic support for cell survival and optic nerve regeneration will be also evoked since all information provided by these studies may be useful for cell replacement strategies.

## RGC Disorders and Associated-Optic Neuropathies

A wide variety of mechanisms, e.g., traumatic, inflammatory, ischemic, or infectious leads to optic neuropathies (Levin and Gordon, 2002). In this chapter, we will focus on the glaucoma-associated optic neuropathy as the leading cause of irreversible blindness and some sporadic inherited optic neuropathies with no current treatment.

### Glaucoma-Associated Optic Neuropathy

According to the literature, the global prevalence of glaucoma in a population aged 40–80 years is 3.54% worldwide (Tham et al., 2014) and the number of patients is estimated to be more than 60 million. Commonly, glaucoma develops initially without self-detection of visual deficit and detectable visual field defects appear at an advanced-stage of the disease. Nevertheless, earlier diagnosis is still possible, looking at the *fundus oculi*, since RGC loss manifests as optic nerve head modification even without self-detection of visual deficit.

Glaucoma can be separated into open-angle and angle-closure glaucoma depending on the morphology of the anterior chamber. Some inherited forms of primary open-angle glaucoma (Allingham et al., 2009) have been reported to be associated with expression of a specific variant or mutation in different genes such as *sine oculis-related homeobox 6* (*SIX6)* (Carnes et al., 2014) or *OPTINEURIN* (*OPTN*) (Rezaie et al., 2002). The common feature of all forms of glaucoma is the progressive degeneration of the optic nerve and loss of RGCs detectable by morphological features, i.e., the reduction of the retinal nerve fiber layer, thinning of the neuroretinal rim of the optic disk and cupping of the optic disk (Alhadeff et al., 2017; Jonas et al., 2017). Different risk factors have been reported such as aging, ethnic background, high myopia and family history (Jonas et al., 2017), but the best-characterized risk factor is an excessive IOP. The relation between high IOP and RGC death is not fully understood but many authors agree to incriminate a mechanical stress to the *lamina criblosa* in the optic nerve head, as a critical site of axonal damage (Chidlow et al., 2011). Mechanical constraints may affect both anterograde and retrograde axonal transport resulting in the degeneration of RGC axons (Pease et al., 2000; Salinas-Navarro et al., 2010; Almasieh et al., 2012). Axonal transport failure has been reported in experimental or genetic models of glaucoma such as DBA/2J mice (Anderson and Hendrickson, 1974; Chihara and Honda, 1981; Pease et al., 2000; Kim et al., 2004; Martin et al., 2006; Balaratnasingam et al., 2007; Crish et al., 2010; Dengler-Crish et al., 2014). One hypothesis points to the impairment of neurotrophic factor delivery compromising RGC survival, as a consequence of the axonal transport blockade caused by excessive IOP (Pease et al., 2000; Salinas-Navarro et al., 2010; Almasieh et al., 2012; Fahy et al., 2016; Kimura et al., 2016). However, glaucoma can take place without abnormal IOP, especially in the case of primary open-angle form and some patients display evolving RGC degeneration despite the normalization of IOP (Sluch and Zack, 2014; Demer et al., 2017). Finally, some people display high IOP without any symptoms of the disease (Friedman et al., 2004), suggesting that other mechanisms may exist and/or that the degenerative process persists despite the abolition of the initial cause of the disease.

**FIGURE 1:**
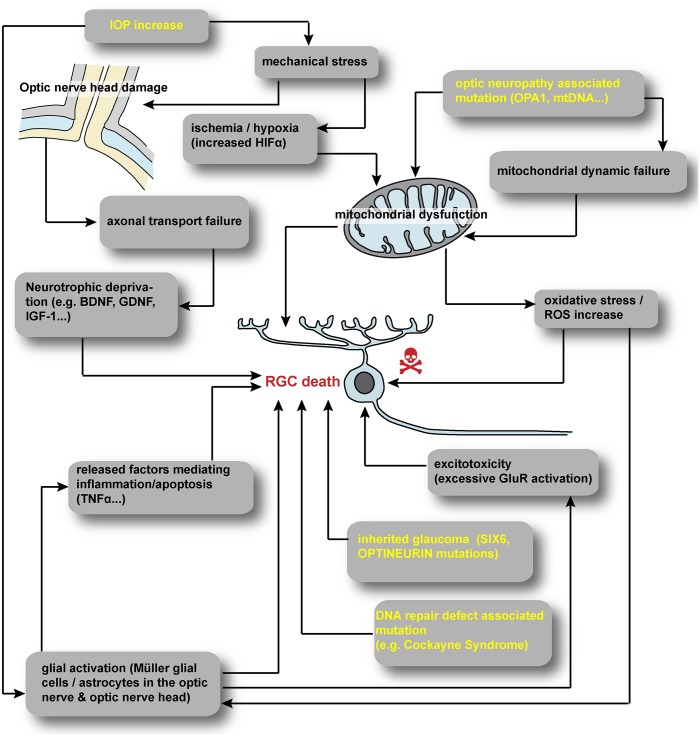
Summary of hypothesized scenarios of RGC degeneration. A vast number of extrinsic and intrinsic cues have been reported to trigger RGC degeneration. Increased IOP in glaucoma may induce mechanical stress at the optic nerve head leading to hypoxia and axonal transport defect. Inherited optic neuropathies often involve directly or indirectly mitochondrial defect. In all RGC disorders, glial cells are suspected to contribute to RGC degeneration. IOP, intraocular pressure; HIF, hypoxia inducible factor; ROS, reactive oxygen species; mtDNA, mitochondrial DNA; GluR, glutamate receptor.

A vast number of extrinsic and intrinsic signals have been reported to trigger RGC death by apoptosis during glaucoma (**Figure 1**). Oxidative stress, hypoxia, excitotoxicity or trophic factor deprivation have been extensively detailed (Qu et al., 2010; Almasieh et al., 2012; Munemasa and Kitaoka, 2013). Intrinsically, the role of specific neurotrophic factors for RGC development and survival is widely accepted (Cellerino et al., 1997; Ma et al., 1998; Almasieh et al., 2012; Harvey et al., 2012; Marler et al., 2014; Kimura et al., 2016). The promotion of RGC survival by Brain-Derived Neurotrophic Factor (BDNF) is well documented both *in vitro* (Johnson et al., 1986; Barres et al., 1988; Meyer-Franke et al., 1995) and *in vivo* after RGC injury (Mansour-Robaey et al., 1994; Sawai et al., 1996; Di Polo et al., 1998; Yip and So, 2000; Almasieh et al., 2012; Harvey et al., 2012). It is widely accepted that the blockage of both anterograde and retrograde axonal transports may disrupt the delivery of neuroprotective factors (Pease et al., 2000; Salinas-Navarro et al., 2010; Almasieh et al., 2012; Fahy et al., 2016). In addition to BDNF, many other neurotrophic factors such as Nerve Growth Factor (NGF), Glial cell-Derived Neurotrophic Factor, Insulin-like Growth Factor-1 (IGF-1) or Leukemia Inhibitory Factor (Yan et al., 1999; Kermer et al., 2000; Mao et al., 2008; Leibinger et al., 2009; Kimura et al., 2016), have been reported to delay or prevent RGC death. In this context, acute glial cell activation is involved in neuroprotection via the delivery of trophic support but depending on the kinetic of glial activation. Acute activation of glial cells is believe to mediate neuroprotection via the delivery of trophic support but conversely chronic gliosis may be essentially neurotoxic via inflammatory mechanisms (Almasieh et al., 2012; Munemasa and Kitaoka, 2013; Vecino et al., 2016).

### LHON and DOA

Inherited optic neuropathies represent a group of genetic disorders causing visual loss characterized by the degeneration of the optic nerve, usually bilateral, associated to the death of RGCs. These pathologies are classified according to the mode of transmission and their non-syndromic (isolated) or syndromic features. Many hereditary optic neuropathies, including the most frequent non-syndromic ones, LHON and DOA, are related to the impairment of mitochondrial function (Yu-Wai-Man et al., 2011; Newman, 2012; Carelli et al., 2017) (**Figure 1**). LHON is a mitochondrial disorder affecting predominately children or young adult, characterized by a rapid and severe visual loss, with rare partial recovery. The prevalence is variable according to the geographic zone ranging from 1 in 30 000 to 1 in 100 000 (Milea and Verny, 2012; Carelli et al., 2017; Jurkute and Yu-Wai-Man, 2017). Three primary mutations, associated with a weak penetrance, have been reported in the mitochondrial genome – revealed by a maternal transmission – which account for approximately 90% of all cases, and all located in genes encoding subunits of the complex I of the respiratory chain (Wallace et al., 1988; Yu-Wai-Man et al., 2011; Carelli et al., 2017; Jurkute and Yu-Wai-Man, 2017). The pathogenic mechanism is mainly due to a reduced energetic efficiency, an increased production of reactive oxygen species, and a disruption of anti-apoptotic pathways (Carelli et al., 2017). DOA is also a mitochondrial disorder starting frequently during early childhood but usually associated with a less severe visual impairment than LHON. Its prevalence is similar to that of LHON and the most frequent mutation is located in the *OPA1* gene (Alexander et al., 2000; Delettre et al., 2000). *OPA1* gene is located in the nuclear genome and encodes for a dynamin-related GTPase addressed to the mitochondrial inner membrane. This protein has been implicated in many functions including mitochondria dynamics, oxidative phosphorylation and apoptosis (Newman, 2012; Chun and Rizzo, 2016; MacVicar and Langer, 2016). Mitochondria display an asymmetric distribution, abundant in the unmyelinated segment of RGC axons in the retinal nerve fiber layer and far less numerous in myelinated parts after crossing the *lamina criblosa*. This specific distribution is tightly linked to the mitochondrial dynamics involving OPA1 function (Yu-Wai-Man et al., 2011; Carelli et al., 2017), suggesting that a disruption of this dynamic process may account for the pathogenesis associated to *OPA1* gene mutations.

Although Cockayne syndrome related to mutations in genes involved in DNA repair cannot be considered as an optic neuropathy disease, phenotypic analysis of several cases showed a loss of RGCs and degeneration of the optic nerve in some patients (Weidenheim et al., 2009). These observations suggest that the loss of RGCs could be also related to gene mutations affecting DNA repair mechanisms but reflects a more global neuronal toxicity taking place in these patients, where a severe neuropathy affecting many regions of the central nervous system is observed. However, in progeroid mouse models, data on retinal changes are rare and when observed cell degeneration is restricted to photoreceptors and/or RPE (Harkema et al., 2016), similar to situations observed in Retinitis Pigmentosa or Age-related Macular Degeneration.

## Retinal Development

Based on our knowledge of retinal development in animal models, a large number of protocols used for the generation of retinal cells have tried to recapitulate “*in the dish*” the major developmental steps required for specification, differentiation and maturation of the retina.

During gastrulation, the eye formation initiates with a series of patterning events governed by specific signals that lead to the specification of a group of neuroepithelial cells within the midline of the anterior neural plate, corresponding to the eye field territory. Delimitation of this territory depends on activation of Fibroblast Growth Factor (FGF) and IGF-1 signaling pathways and repression of both Transforming Growth Factor beta (TGFβ)/Bone Morphogenetic Protein (BMP) and Wnt pathways (Graw, 2010; Zuber, 2010). This region expresses several eye-field transcription factors (EFTFs), including different homeodomain-containing factors, such as Paired box 6 (Pax6), T-box transcription factor TBX3, LIM homeobox protein 2 (Lhx2), Orthodenticle homeobox 2 (Otx2), Retina and anterior neural fold homeobox (Rax), Six3 and Six6. These transcription factors act synergistically to form and maintain the eye field territory in a self-regulating feedback highly conserved between species (Zuber, 2010). Recent experiments in xenopus by injection of blastomeres of 2-cell staged embryos demonstrated that Tbx3 and Pax6 are the only EFTFs sufficient to determine pluripotent cells to a retinal lineage (Motahari et al., 2016).

This EFTF-expressing region expands bilaterally during midline formation to form two optic areas, where the evagination of the neuroepithelium leads to the optic vesicle formation. The lens placode invaginates into the optic vesicle resulting in the formation of the lens vesicle. Simultaneously, the optic vesicle invaginates to form the optic cup with an outer and inner layer committed, respectively, into the RPE and the neural retina lineage under the influence of exogenous signals coming from adjacent tissues, such as FGF, Sonic hedgehog (Shh) or agonists of the TGFβ/BMP pathway (Fuhrmann, 2010; Fuhrmann et al., 2014). At this time, the future neural retina consists of immature multipotent retinal progenitor cells (RPCs), that have the ability to give rise to all retinal cell types in an overlapping chronological order, generally conserved among many species. Early-born cell types include retinal ganglion, amacrine and horizontal cells and cone photoreceptors while rod photoreceptors, bipolar and Müller glial cells are generated mainly at later stages (Young, 1985; Turner and Cepko, 1987). The competence model is widely accepted and it supports the idea for the generation of neuronal diversity, where intrinsic factors control the temporal identity of RPCs, a period during which they are able to generate different cell types but only within a specific subset (Boije et al., 2014; Cepko, 2014; Goetz et al., 2014). Ikaros and Casz1 have been proposed as factors that contribute to the establishment of the temporally restricted progenitor cell fates in developing murine retina (Elliott et al., 2008; Mattar et al., 2015). Several lines of evidence demonstrate that a hierarchical gene regulatory network with basic-loop-helix (bHLH)-type and homeodomain-containing factors is at the basis of specific retinal cell fate acquisition made by RPCs (Boije et al., 2014). The expression of transcription factors influencing specific cell fate such as bHLH transcription factor atonal homolog 7 (Atoh7, also known as Math5), Forkhead box N4 (FoxN4) and Pancreas specific transcription factor 1a (Ptf1a) is inhibited by Visual system homeobox 2 (Vsx2) which is largely expressed in proliferative RPCs. When Vsx2 expression diminished during the development, each RPC is committed to a different cell lineage: RGC lineage governed by Atoh7 and amacrine and horizontal cell lineages by transitory expression of Atoh7 and the presence of FoxN4 and Ptf1a. Later, the loss of Atoh7 expression in RPCs provides a permissive environment for a photoreceptor cell fate in absence of both FoxN4 and Ptf1a (Bassett and Wallace, 2012; Boije et al., 2014).

Retinal ganglion cells are the first retinal cell type to be specified and the basic helix-loop-helix transcription factor Atoh7 is a major determinant of RGC commitment. Atoh7 expression is required for RPCs to acquire competence for an RGC precursors fate (Brown et al., 2001; Wang et al., 2001) and its overexpression promotes cell cycle exit and enhances production of RGCs (Zhang et al., 2018). Atoh7 controls the expression of both POU domain, class 4, transcription factor 2 (POU4F2, as known as Brn3b) and homeobox transcription factor insulin gene enhancer protein ISL-1 (Isl1), two transcription factors required for controlling the initiation of the whole RGC transcriptional program (Wu et al., 2015). Mutations or knockdown of these genes result in failure or disruption of RGC development in animal models (Gan et al., 1996; Brown et al., 2001; Kay et al., 2001; Wang et al., 2001; Mu et al., 2008). Downstream RGC-specific gene network is involved in RGC cell subtype specification and/or RGC maturation, as recently shown for POU domain, class 4, transcription factor 1 (POU4F1, as known as Brn3a), and 3 (POU4F3, as known as Brn3c), Ebf helix-loop-helix transcription factors (Ebf1 and 3) and Onecut 1 and 2 (Gan et al., 1996; Badea et al., 2009; Jin et al., 2010; Sapkota et al., 2014). Conditional knock-out of Sox4 and Sox11, two members of Sox C gene family, led to a moderate to a complete loss of RGCs in double Sox4/Sox11-null retinas (Jiang et al., 2013). These SoxC genes have been reported as essential contributors to RGC development implicated in intermediate position between Atoh7 and POU4F2 (Jiang et al., 2013) and to participate to the control of axon projections (Kuwajima et al., 2017). Recent transcriptome analysis by RNA sequencing of genetically labeled RGC targeting the three POU4F transcription factors allowed the identification of combinatorial molecular codes (transcription factors and cell surface molecules) expressed in different RGC subtypes (Sajgo et al., 2017).

Main extrinsic factors known to regulate RGC neurogenesis are members of the FGF family and Shh. Elegant experiments in fish and chick using specific FGF mutants or pharmacological inactivation of FGF signaling demonstrated that secreted FGF3 and FGF8 from cells located into the organizing center, such as optic stalk, coordinate the progression of RGC development (Martinez-Morales et al., 2005). This propagation of RGC neurogenesis is also controlled by Shh signaling, since an arrest of RGC differentiation has been observed in zebrafish shh mutant (Neumann and Nuesslein-Volhard, 2000). Interestingly in mouse and chick retina, the Shh pathway has been described as a negative feedback regulator of RGC neurogenesis, where nascent RGCs secreted Shh and modulate RGC differentiation within a normal period of retinogenesis (Zhang and Yang, 2001; Wang et al., 2005).

During differentiation, RGCs extent their axons on the retinal surface, toward the optic cup, to exit the eye. Axon growth in the optic fiber layer is mainly under the control of inhibitory signals such as Slit1/2 preventing aberrant extension into the retina (Herrera and Erskine, 2017). Then, the axons fasciculate and continue to grow in order to reach the optic chiasm where a complex set of cues including both transcription factors and extrinsic signals leads the axon to cross or not the midline, forming the contralateral and ipsilateral projections (Assali et al., 2014; Herrera and Erskine, 2017). Finally, RGC axons have to reach different brain targets including lateral geniculate nuclei, superior colliculi and other accessory visual structures; topographic mapping and synaptic refinement are both strongly activity-dependent and continue with visual experience (Priebe and McGee, 2014; Arroyo and Feller, 2016).

## Pluripotent-Stem Cells-Derived Rgcs

### Generation of RGC From Mouse PSCs

One of the first robust protocols for the generation of retinal cells used factors known to pattern anterior neural and retinal fate *in vivo* (Ikeda et al., 2005). In this protocol, 3D serum-free floating embryoid bodies (SFEB) were generated from mouse ESCs with the addition of specific factors, Lefty-A (a Nodal antagonist) and Dickkopf-1 (Dkk-1), a Wnt pathway antagonist. Just before plating the cells, the addition of serum and Activin-A induced a significant generation of RPCs co-expressing Rx and Pax6. Interestingly, 9% of cells in SFEBs expressed the specific RGC marker Islet1. Based on this protocol, the group of M. Takahashi, using a mouse *Rx*-reporter ESC line demonstrated that Rx-positive SFEBs displayed an RGC population co-expressing Pax6 and Islet1 (10.1%) after blockade of NOTCH pathway (Osakada et al., 2008).

A full adherent culture system (2D) relies on the direct differentiation of mouse PSCs into RPCs including RGCs, where PSCs were directly cultured into gelatin-coated plates without any preliminary 3D embryoid bodies (EB) formation. In this context, using a similar medium as previously described (Osakada et al., 2008), the overexpression of a specific RGC marker, *Atoh7,* promoted the differentiation of mouse iPSCs into retinal ganglion-like cells, displaying long synapses and specific expression patterns including Atoh7, Isl1, Brn3b, and Thy1.2 (Chen et al., 2010). Other groups have adapted this protocol to generate RGCs from mouse PSCs by overexpressing RPC markers such as NeuroD1 (Huang et al., 2018) or Pax6 (Kayama et al., 2010). The co-culture of adherent mouse ESCs with adult mouse retina tissue improved their differentiation toward the retinal lineage as well as the generation and maturation of RGCs (Aoki et al., 2007, 2008).

A pioneer paper by the group of Y. Sasai demonstrated the self-organized generation of 3D neuro-retinal structures from mouse ESCs, recapitulating the overall retinal induction (Eiraku et al., 2011). The quick re-aggregation of SFEB in presence of knockout serum (KSR) and Matrigel promoted the formation of optic cup-like structures containing invaginated neural retina of a rigid continuous stratified epithelium, directly self-organized in an apical-basal manner. This epithelium recapitulated the stepwise acquisition of domain-specific properties, including RGCs (Brn3-, Pax6- and Calretinin-positive cells) that are localized in the innermost region.

Different groups have developed 3D/2D stepwise protocols (**Table 1**) by first promoting the neural and retinal induction of 3D mouse iPSC-derived EBs before transferring the RPCs obtained in an adherent culture system (2D), which is crucial for promoting RGC axonal growth (Jagatha et al., 2009; Xie et al., 2014; Parameswaran et al., 2015; Tanaka et al., 2016; Teotia et al., 2017). The iPSC-derived EBs cultured in a classical neural induction medium (Ikeda et al., 2005) supplemented with fibronectin and Noggin generated a RPC pool. These cells were then plated on poly-D-lysine/laminin coated dishes in the same medium in presence of FGF2 to favor the enrichment in RPCs. In some of these protocols, the addition of specific factors, such as Shh, FGF8, DAPT (an inhibitor of Notch signaling pathway), follistatin and cyclopamine, followed by treatment with BDNF, Forskolin, cAMP and Ciliary neurotrophic factor (CNTF) in specific time windows promoted, respectively, the generation and the maturation of RGC. The efficiency of these protocols was assessed by the expression of different RGC markers like Brn3b, Rpf1, Isl1, and Thy1 and the functional maturation, using electrophysiological approaches. An interesting strategy to obtain adequate amounts of RGC neurites, was performed by Maekawa et al. (2015) whom directly replated the optic vesicles derived from EBs on matrigel-coated plates, rather than dissociated the 3D structures.

**Table 1:** Retinal ganglion cell (RGC) differentiation protocols from mouse pluripotent stem cells.

Culture system 3D/2D	Reference	PSC type	Neural retina induction medium	RGC differentiation conditions	RGC identification	RGC isolation
3D	Eiraku et al., 2011	ESC	GMEM + 1.5% KSR + pyruvate + mercaptoethanol + NEAA + Matrigel	N.A.	Brn3a, Pax6, Calretinin	N.A.
	DiStefano et al., 2018	ESC/iPSC	DMEM-F12 + Glutamax + N2 + B27 + mercaptoethanol + taurine + 9-*cis* retinal + 2% FBS + IGF-1	N.A.	Brn3a	N.A.
3D (EB) → 2D	Ikeda et al., 2005	ESC	GMEM + 5% KSR + pyruvate + mercaptoethanol + NEAA + Lefty-A + FCS + DKK1 + Activin-A	Medium: no modif^∗^ Coating: PDL/laminin/fibronectin	Islet1	N.A.
	Osakada et al., 2008	ESC	GMEM + 5% KSR + pyruvate + mercaptoethanol + NEAA + Lefty-A + 5% FBS + DKK1 + Activin-A	Medium: no modif^∗^ Coating: PDL/laminin/fibronectin	Pax6, Islet1	N.A.
	Jagatha et al., 2009	ESC	DMEM-F12 + N2 + 0.5% FBS + heparin + FGF2	Medium: DMEM-F12 + N2 + 0.5% FBS + FGF2 Coating: PDL/laminin	Ath5, Brn3b, RPF-1, Thy-1 and Islet-1,	N.A.
	Xie et al., 2014	iPS reporter cell line: Atoh7-Cre/ROSA YFP knock in line	DMEM-F12 + 10% FBS + N2 + B27 + NEAA + sodium pyruvate + CKI-7 + SB431542 + DAPT	Medium: DMEM-F12 + N2 + B27 + NEAA + sodium pyruvate + CKI-7 + SB431542 + DAPT Coating: Matrigel	Brn3a, NF68	N.A.
	Parameswaran et al., 2015	iPSC	DMEM-F12 + N2 + B27 + insulin + transferrin + sodium selenite + glutamine + fibronectin + Noggin + FGF2	Medium: DMEM-F12 + N2 + glutamine + SHH + FGF8 + DAPT + follistatin + cyclopamine Coating: PDL/laminin	Brn3b, Rpf1, Islet1, Thy1	THY1.2 magnetic beads purification
	Maekawa et al., 2015	ES reporter cell line: Follistatin4::Venus mice line	Neurobasal-A + B27 + L-glutamine + Retinoic acid + L-taurine	Medium: no modif^∗^ Coating: 100% Growth factor reduced - Matrigel	BRN3a, Brn3b, Fstl4, SMI312, βIII tubulin	N.A.
	Tanaka et al., 2016	ESC/iPSC	DMEM-F12 + GlutaMAX + N2	Medium: DMEM-F12 + Glutamax + N2 + BDNF + Retinoic acid + (1–10%) FBS Coating: PDL/laminin	Brn3, Math5, βIII tubulin, Sncg, Islet1, Tau, NFM, NFH, NFL	N.A.
	Teotia et al., 2016	ES modified cell line: shRNA-mediated REST loss of function	DMEM-F12 + N2 + B27 + glutamine + Noggin + DKK1 + FGF2 + IGF-1	Medium: DMEM-F12 + N2 + B27 + glutamine + fibronectin + Noggin + FGF2 + SHH + FGF8 + DAPT + follistatin + cyclopamine + BDNF + CNTF + Forskolin + cAMP + Y27632 + NT4 Coating: PDL/laminin	Atoh7, Brn3b, Islet1, βIII tubulin	N.A.
2D	Aoki et al., 2007	ESC	A-MEM + 10% FCS Co-culture with adult mouse retina ± NMDA injection	Medium: no modif^∗^ Adherent condition: PA6 stromal cells	Hu, Brn3b	N.A.
	Chen et al., 2010	iPSC	GlutaMAX + 15% FBS + FGF2 + NEAA + N2 + B27 + DKK1 + Noggin + DAPT	Medium: Neurobasal + 3% FBS + N2 + B27 + GlutaMAX + DKK1 + Noggin + DAPT + NEAA Coating: Gelatin 0.1% Overexpression: Math5	Math5, Isl1, Brn3b, Thy1	N.A.
	Kayama et al., 2010	ES reporter cell line: Pax6 reporter cell line	DMEM-F12 + N2 + 0,05% fibronectin	Medium: no modif^∗^ Coating: Gelatin	Six3, Shh, Brn3a, Brn3b, Thy1, Math5, Pax6, Islet1, melanopsin	N.A.
	Huang et al., 2018	iPSC	Neurobasal + 10% FBS + L-glutamine + NEAA + N2 + B27	Medium: no modif^∗^ Coating: Gelatin 0.1% Overexpression: NeuroD1	Brn3b, Islet1, Math5, Thy1.2	N.A.

*A-MEM, Minimum Essential Medium Eagle with Alpha modification; BDNF, brain-derived Neurotrophic Factor; FGF2, beta fibroblast growth factor; cAMP, cyclic adenosine monophosphate; CNTF, Ciliary Neurotrophic Factor; DAPT, N-[N-(3,5-Difluorophenacetyl)-L-alanyl]-S-phenylglycine t-butyl ester; DKK1, Dickkopf-1; DMEM-F12, Dulbecco’s Modified Eagle Medium: Nutrient Mixture F-12; EB, embryoid bodies; ESC, embryonic stem cells; FBS, fetal bovine serum; FCS, fetal calf serum; FGF2, fibroblast growth factor 2; FGF8, fibroblast growth factor 8; GMEM, Glasgow Modified Eagle Medium; IGF-1, insulin growth factor-1; iPSC, induced pluripotent stem cells; KSR, knockout serum replacement; NEAA, non-essential amino acids; NT4, neurotrophin-4; PDL, poly-D-lysin; REST, repressor element-1 silencing transcription factor; RWV, rotating-wall vessel; SHH, Sonic Hedgehog; shRNA, short hairpin RNA; N.A., not applicable; No modif^∗^, same medium as the corresponding neuro-retinal medium.*

In these protocols, different strategies have been used to isolate PSC-derived RGCs, such as the use of atoh7 reporter cell line (Xie et al., 2014), or by targeting the Thy1 glycoprotein (Parameswaran et al., 2015), a well-known RGC specific surface marker in the retina (Barres et al., 1988).

To go further in disease modeling and future regenerative medicine applications, a recent 3D culture system was developed to culture retinal organoids derived from mouse PSCs in rotating-wall vessels (RWV) bioreactors (DiStefano et al., 2018). This bioprocess recapitulated spatiotemporal development and maturation of retinal organoids in an accelerated manner (25 days) in comparison with static culture conditions (32 days), similar to the development of postnatal day 6 mouse retina *in vivo*. Brn3a-expressing RGCs were detected at the basal side of the neural retina organoids as earlier as 15-days.

### Generation of RGCs From Human PSCs

Original protocol was described by the group of T. Reh, where neural induction was induced in EB suspension (similar to SFEB system), by a combination of the key factors Noggin (a BMP antagonist), Dkk-1 and IGF-1 (Lamba et al., 2006). After seeding the cells onto coated poly-D-lysine/Matrigel plates, this cocktail of factors supplemented with FGF2 and pro-neural supplements was used for further differentiation. Among early retinal cell types generated, RGCs were identified by several specific markers, such as PAX6, HuC/D, Neurofilament-M and βIII tubulin. Instead of making EB, plating the clumps of human ESCs directly on Matrigel with the same differentiation media led to similar retinal differentiation in complete adherent cell culture conditions (Lamba et al., 2010). This pioneer “Lamba protocol” led to other protocols (**Table 2**) improving the generation of RPCs and mature RGCs (Gill et al., 2014). Retinal structures containing RGCs identified by Brn3b and Neurofilament-200 have been obtained in similar adherent conditions in absence of Matrigel by addition of Noggin, FGF2, DKK-1, IGF-1, and FGF-9 in specific time windows (Singh et al., 2015). Another recent study, based on “Lamba protocol” (Lamba et al., 2010), validated a simple 2D-adherent differentiation protocol of human iPSC-derived neural rosettes (Teotia et al., 2017), similar to protocol developed using mouse PSCs (Parameswaran et al., 2010, 2015). One strategy combining EBs and subsequent human PSC-derived neurosphere cultures obtained from EB-derived neural rosettes has been developed to enhance RGC differentiation (Riazifar et al., 2014). Interestingly the final culture of neurospheres in laminin-coated plates in presence of serum and DAPT led to approximately 30% of the cells expressing a whole range of RGC markers (BRN3A, BRN3B, THY1, ISLET1, γ-SYNUCLEIN, and ATOH7) (Riazifar et al., 2014).

**Table 2:** Retinal ganglion cell differentiation protocols from human PSCs.

Culture system 3D/2D	Reference	PSC type	Neuro-retinal induction medium	RGC maturation conditions	RGC identification	RGC isolation
3D	Nakano et al., 2012	ESC	Induction: GMEM + 20% KSR + NEAA + pyruvate + mercaptoethanol + 10% FBS + Matrigel-growth factor reduced + SAG + CHIR99021 Neuro-retinal culture: DMEM-F12 + N2 + GlutaMAX	Medium: no modif^∗^ Coating: N.A.	Brn3a, Rxrγ	N.A.
	Reichman et al., 2017	iPSC	Induction: DMEM-F12 + N2 Maturation: DMEM-F12 + B27 + NEAA	Medium: DMEM-F12 + B27 + NEAA (preliminary datas) Coating: PDL/laminin (preliminary datas)	Brn3a (preliminary datas: Pax6, Thy1, βIII tubulin)	N.A.
3D → 2D	Meyer et al., 2011	iPSC/ESC	DMEM-F12 + B27 + N2 + glutamine + NEAA + Noggin + DKK-1 + IGF-1 + FGF2	Medium: DMEM-F12 + B27 + N2 + glutamine + Shh + FGF8 + DAPT + follistatin + cyclopamine + BDNF + CNTF + Forskolin + cAMP + Y27632 Coating: Matrigel	Brn3, Pax6, βIII tubulin, Calretinin	N.A.
	Zhong et al., 2014	iPSC	DMEM-F12 + B27 + NEAA + 10% FBS + Taurine + GlutaMAX	Medium: no modif^∗^ Coating: Matrigel	Brn3, Hu C/D	N.A.
	Deng et al., 2016	iPSC	Neurobasal + B27 (without VitA) + N2 supp + NEAA + L-glutamine + β-mercaptoethanol	Medium: no modif^∗^ Coating: Matrigel. Overexpression: Atoh7	Brn3b, Tuj, Islet1, Calretinin	N.A.
	Gill et al., 2016	ESC	DMEM-F12 + GlutaMAX + 10% KSR + B27 + Noggin + IGF-1 + DKK1	Medium: DMEM-F12 + N2 + B27 + GlutaMAX + Noggin + IGF-1 + DKK1 + FGF2 Coating: Matrigel/PDL	NFM, βIII tubulin, Brn3a, Hu C/D	CD90-coupled magnetic microbeads (MACS)
	Langer et al., 2018	iPSC/ESC	DMEM-F12 + B27 + N2 + glutamine + NEAA + Noggin + DKK-1 + IGF-1 + FGF2	Medium: DMEM-F12 + B27 + N2 + glutamine + Shh + FGF8 + DAPT + follistatin + cyclopamine + BDNF + CNTF + Forskolin + cAMP + Y27632 Coating: Laminin	Brn3, Islet1, Rbpms, Sncg, βIII tubulin, Smi32, Map2	N.A.
	Kobayashi et al., 2018	iPSC	DMEM-F12 + 10% KSR + BMP4 + N2 + GSK3 inhibitor + VEGFR/FGFR	Medium: Neurobasal + CNTF + BDNF + forskolin + *N*-acetylcysteine + FGF2 + B27 + glutamine + insulin + sodium pyruvate + progesterone + putrescine + sodium selenite + triiodothyronine Coating: PDL/Laminin	Atoh7, Smi32, Brn3b, Islet1, Rbpms, Thy1	Anti-Thy1 antigen (immunopanning)
2D	Lamba et al., 2006	ESC	DMEM-F12 + 10% KSR + Noggin + DKK1 + IGF-1 + FGF2 + B27 + N2	Medium: no modif^∗^ Coating: Matrigel/PDL	Pax6 (RPC population), HuC/D, Tuj1	N.A.
	Sluch et al., 2015	ESC	DMEM-F12 + Neurobasal + GlutaMAX + N2 + B27 + FGF8 + FGF-A + Taurine + 10% FBS	Medium: no modif^∗^ Coating: Matrigel.	Brn3b, Tuj1, Rbpms, Map2	Specific fluorescent reporter expression (FACS)
	Singh et al., 2015	ESC	Neurobasal + N2 + B27 (without RA) + Noggin Induction medium: DKK-1 + IGF-1 Differentiation medium: L-glutamine + β-mercaptoethanol + FGF2 + FGF9 + BSA + amphotericin-B + gentamicin	Medium: no modif^∗^ Coating: Gelatin/Laminin	Brn3b, NF200	N.A.
	Teotia et al., 2016	iPSC	DMEM-F12 + Noggin + DKK-1 + IGF-1 + B27 + N2	Medium: DMEM-F12 + Shh + FGF8 + DAPT + follistatin + cyclopamine + BDNF + CNTF + Forskolin + cAMP + Y27632 Coating: Matrigel.	Atoh7, Brn3, βIII tubulin, Thy1.2	N.A.

*BDNF, brain-derived Neurotrophic Factor; FGF2, beta fibroblast growth factor; BMP4, bone morphogenetic protein 4; BSA, bovine serum albumin; cAMP, cyclic adenosine monophosphate; CNTF, ciliary Neurotrophic Factor; DAPT, N-[N-(3,5-Difluorophenacetyl)-L-alanyl]-S-phenylglycine t-butyl ester; DKK1, Dickkopf-1; DMEM-F12, Dulbecco’s Modified Eagle Medium: Nutrient Mixture F-12; ESC, embryonic stem cells; FACS, fluorescent-activating cell sorting; FBS, fetal bovine serum; FGF2, fibroblast growth factor 2; FGF8, fibroblast growth factor 8; FGF9, fibroblast growth factor 9; FGF-A, fibroblast growth factor-acidic; FGF-R, fibroblast growth factor receptor; GMEM, Glasgow Modified Eagle Medium; IGF-1, insulin growth factor-1; iPSC, induced pluripotent stem cells; KSR, knockout serum replacement; NEAA, non-essential amino acids; NF200, Neurofilament-200; NFM, Neurofilament-M; PDL, poly-D-lysin; RA, retinoic acid; VEGFR, vascular endothelial growth factor receptor; VitA, vitamin A; SAG, Sonic Hedgehog agonist; SHH, Sonic Hedgehog; N.A., not applicable; No modif^∗^, same medium as the corresponding neuro-retinal medium.*

An original protocol allowing the generation of RGCs from adherent human ESCs was recently reported (Sluch et al., 2015). Retinal development recapitulation was confirmed by evaluation of the expression of some EFTFs and the development of a CRISPR-engineered RGC fluorescent reporter cell line, where mCherry was knock-in in the BRN3B locus, led to the identification and the selection of RGCs by FACS. Surprisingly, subsequent isolated RGCs, immunoreactive for different RGC markers (BRN3B/TUJ1, RBPMS) developed neurite networks and displayed physiological properties associated with mature RGCs (Sluch et al., 2015). This particular reporter cell line contributed to the identification of three cell sub-populations, after Thy1 FAC-sorting and single cell RNA sequencing analysis. These sub-populations exhibited different levels of maturity and contained upregulated genes for neuronal outgrowth, neuronal function and axon guidance, from the earliest to the latest cell clusters, respectively (Daniszewski et al., 2018).

Currently, a considerable panel of novel *in vitro* 3D/2D stepwise differentiation protocols (**Table 2**) were designed (Meyer et al., 2011; Nakano et al., 2012; Zhong et al., 2014; Gill et al., 2016). A compelling 3D/2D stepwise differentiation protocol from human PSC lines achieved the isolation of structures displaying the characteristics of optic vesicle (OV)-like structures (Meyer et al., 2011). Self-formed cell aggregates from human iPS colonies were plated to form neural clusters and then manually picked up. RGCs were identified in floating OV-like structures mostly at the periphery of the structures, and characterized by several markers, such as BRN3a/b, βIII-TUBULIN, CALRETININ, and PAX6. This protocol has been nicely improved for the generation of photoreceptors in 3D retinal optic cup (OC)-like structures, recapitulating the developmental organization of retina *in vivo* (Zhong et al., 2014). RGCs, identified as BRN3- and Hu C/D-expressing cells were observed in the innermost zone of the laminated OC-like structures.

Studies focusing on the differentiation of RGCs, adapting this 3D/2D stepwise protocol have been recently reported allowing the identification of different types of RGCs based on morphological, phenotypic, and functional characteristics (Langer et al., 2018). Different RGC subtypes were determined by morphological features and single-cell RNA sequencing analysis confirmed the expression of subtype-specific markers. Among the identified RGC subtypes, the direction-selective ON-OFF RGCs, ON RGCs, α-RGCs and intrinsically photosensitive RGCs were presumptively observed (Langer et al., 2018).

Alternatively and based on previous mouse work, Nakano et al. (2012) initiated a relevant 3D-retinal differentiation approach, with self-forming of OC-like structures derived from human ESCs, based on their similar protocol for mouse ESCs (Eiraku et al., 2011). At early stages of development (around 24 days), the RGC population constituted the first-born cell population that gradually increased to form a distinct layer at the most basal region of the human ESC-derived NR epithelium in 1 week. Many retinal differentiation strategies have been adapted from this pioneer “Nakano protocol” to study functional axonal extensions (Maekawa et al., 2015; Tanaka et al., 2015, 2016; Völkner et al., 2016; Yokoi et al., 2017; Kobayashi et al., 2018).

**FIGURE 2:**
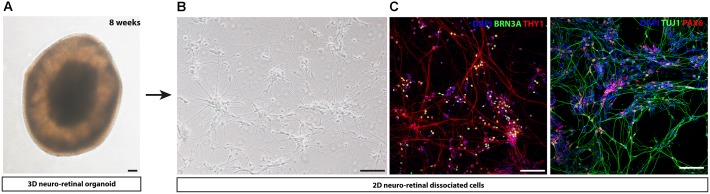
Representative images of human iPS cell-derived RGCs by adapting protocol from Reichman et al. (2017). **(A)** Representative image of a self-organized 8-week-old 3D retinal organoid **(B)** Retinal cells dissociated from 8-week-old 3D retinal organoids observed using phase contrast microscopy. **(C)** Immunostaining on retinal cells dissociated from 8-week-old 3D retinal organoids reveal the expression of neuronal markers (THY1, TUJ1), retinal progenitor cell-associated marker (PAX6) and RGC-associated marker (BRN3A). Nuclei were counterstained by DAPI (blue). Scale bars: 100 μm.

An innovative strategy bypassing EB formation, addition of Matrigel and other exogenous factors has been developed for different human iPSC lines (Reichman et al., 2014). Overgrowing iPSCs in absence of pluripotency factor FGF2 and in presence of proneural supplements such as N2 and/or B27, could generate self-forming neuro-retinal structures. Maintenance of the isolated retinal organoids in long-term floating culture with B27 supplement allowed the differentiation of all retinal cell types including the RGC population, identified by BRN3A immunostaining. This protocol can be adapted in a xeno-free and feeder-free culture conditions (Reichman et al., 2017), compatible to a clinical setting allowing the production of cells of therapeutic interest. One example of 8-week-old retinal organoid is illustrated in **Figure 2A**. As previously described for the different strategies, the survival and maturation of RGCs can be promoted by a subsequent adherent cell culture step, seeding cells onto poly-D-lysine/laminin-coated plates after enzymatic dissociation of 8-week-old retinal organoids (**Figures 2B,C**).

Even though retinal organoids resume the self-generation of 3D structures with specific retinal cell types, some limitations exist to study the maturation of RGCs. Indeed, it has been clearly reported that the percentage of RGC gradually decreased *in vitro* as the culture was extended (Meyer et al., 2011; Nakano et al., 2012; Reichman et al., 2014). Floating culture of retinal organoids is a strong hindrance to axon growth outside the structure and the lack of projection targets should prevent the normal development of RGC and may induce their death, probably by apoptosis. Brain organoid technologies derived from pioneer work from (Lancaster et al., 2013) allow now the generation of organoids that can model the development of different human brain regions (Di Lullo and Kriegstein, 2017). Recent work from the group of P. Arlotta analyzed gene expression in over 80,000 individual cells from human brain organoids and found the presence of different clusters of neuronal-specific genes identified at 6 months of differentiation, including a cluster of retinal population-specific genes (Quadrato et al., 2017). Subclustering of the retinal cluster revealed that RGCs were observed in 55% of organoids, but no clear fasciculation of axons was observed, asking the question of the ability of RGC to form an optic nerve-like structure. Fusing retinal and brain organoids could be a way to model the specific optic nerve circuitry. An alternative strategy could be the development of specific device for axon fascicle formation allowing the formation of a RGC nerve organoid, as recently reported for hPSC-derived motoneurons (Kawada et al., 2017).

For the translation of iPSC technology to the clinics, transplantable cells should be purified into a homogenous RGC population isolated from mitotically active cells or residual undifferentiated iPSCs that could be teratogenic. As genetic engineering or viral labeling of the cells are not suitable for clinical applications, the identification of cell surface markers characterizing human PSC-derived RGCs is important. As for mouse RGCs isolated from post-natal retina (Barres et al., 1988) or derived from mouse PSCs (Parameswaran et al., 2015), the CD90 (THY1) cell surface marker has been recently used to select human PSC-derived RGCs (Gill et al., 2016; Kobayashi et al., 2018).

Based on “Lamba protocol” (Lamba et al., 2006), Gill et al. (2016) used a magnetic-activated cell sorting (MACS) strategy, with microbeads coupled with a CD90 antibody and demonstrated that MACS enrichment yielded 77 ±9% THY1-positive cells, while less than 5% of the differentiating EB-dissociated cells (around 30–45 days of differentiation) expressed THY1 in the culture. Transcriptome analysis and electrophysiological recordings revealed similarity of enriched human ESC-derived RGCs to RGCs *in vivo* (Gill et al., 2016). Another selection strategy of RGCs derived from human iPSCs, adapted from the “Nakano protocol” (Nakano et al., 2012), was achieved with a CD90-based immunopanning of the retinal cell population (Kobayashi et al., 2018). Surprisingly, the BRN3B-positive RGCs collected at older stages of maturation (around 90–110 days) extended longer neurites compared to RGCs at earlier stages (70–90 days).

All these data reporting, selection, maturation and/or functional assessment of human PSC-derived RGCs should allow to study patient-derived iPSC lines, for a better understanding of ocular disease related to RGC impairment. In this context, different groups have already derived iPSCs from somatic cells of patients diagnosed for different optic neuropathies (Chen et al., 2016; Ohlemacher et al., 2016; Teotia et al., 2017; Wong et al., 2017) (**Table 3**). For example, the generation of LHON-patient iPSC allowed the exploration of oxidative phosphorylation defect (Hung et al., 2016). In a more recent study, the same group demonstrated that replacing LHON mitochondrial DNA using cybrid technology in patient iPSCs can prevented the death of iPSC-derived RGCs (Wong et al., 2017). Another iPSC line carrying mutation in the *Optineurin* (*OPTN*) gene has been recently derived (Ohlemacher et al., 2016) and higher cell death of iPSC-derived RGCs carrying the mutation has been observed. iPSCs derived from a DOA-patient carrying an *OPA1* mutation was also reported and these mutated iPSCs were unable to correctly differentiate into RGCs and exhibited signs of apoptosis compared to free-mutation iPSCs (Chen et al., 2016).

**Table 3:** Published PSC lines carrying mutation for optic neuropathies and disease modeling studies/PSC-derived pathological models for optic neuropathies.

Clinical diagnosis	Gene	Mutation	Cell type origin	Reprogrammation	Differentiated cells	Reference
Glaucoma	OPTINEURIN	E50K	PBMCs (Blood)	Sendai Virus (OKSM)	Neurons	Minegishi et al., 2013
Glaucoma	TBK1	780 kb duplication 12q14	Fibroblasts	Sendai Virus (OKSM)	RGCs	Tucker et al., 2014
Glaucoma	OPTINEURIN	E50K	Fibroblasts	mRNA (OKSM)	RGCs	Ohlemacher et al., 2016
Glaucoma	CYP1B1	c.1403_1429dup	Fibroblasts	Sendai Virus (OKSM)	N.A.	Bolinches-Amorós et al., 2018
Glaucoma	SIX6	rs33912345; C > A; His141Asn	PBMCs (Blood)	Retrovirus (OKSM)	N.A.	Teotia et al., 2017
DOA	OPA1	intron24 c.2496+1 G > T	Fibroblasts	Retrovirus (OKSM)	RGCs	Chen et al., 2016
DOA	OPA1	c1861 C > T; p.Gln621Ter	Fibroblasts	Sendai Virus (OKSM)	N.A.	Galera-Monge et al., 2016
DOA	OPA1	c.610+364G > A, c.1311A > G het.	Fibroblasts	Episomal (OKSM)	N.A.	Hauser et al., 2016
LHON	MT-ND6	14484 T to C	Fibroblasts	Retrovirus (OKSM)	RPE	Zahabi et al., 2012
LHON	MT-ND4	m.11778G > C	Fibroblasts	Episomal (OKSM/Lin28/ shRNAp53)	RGCs	Hung et al., 2016; Wong et al., 2017
	MT-ND1/MT-ND6	m.4160T > C/m. 14484T > C	Fibroblasts			
LHON	MT-ND4	m.11778G > A	PBMCs (Blood)	Sendai Virus (OKSM)	N.A.	Lu et al., 2018
LHON	MT-ND4	m.11778G > A	PBMCs (Blood)	Sendai Virus (OKSM)	RGCs	Wu et al., 2018
Optic atrophy (Wolfram syndrome)	CISD2	c.103+1 G > A (hom; & het.)	Fibroblasts	Episomal (OKSM/Lin28)	N.A.	La Spada et al., 2018

## Optic Neuropathies and Cell Therapy: Cell Replacement Strategies

Two major strategies can be developed to regenerate the optic nerve. One strategy aims to promote survival of remaining RGCs and regrowth of residual axons; the other strategy deals with the replacement of lost RGCs derived from different cell types, mainly from PSCs. The second one is probably more challenging but should have the advantage to be suitable for patients with very few remaining endogenous RGCs and both strategies are not exclusive. Co-administration of neurotrophic factors with transplantation of PSC-derived RGCs could be envisaged to favor both survival and axonal growth of both endogenous and exogenous RGCs.

With regards to glaucoma, some strategies aiming at transplanting mesenchymal stem cells (MSCs) and/or stem cell-derived trabecular meshwork cells with the questionable purpose to improve the drainage of aqueous humor and the control of IOP (Chamling et al., 2016; Yun et al., 2016; Zhu et al., 2016). This review is essentially focused on cell therapy approaches for the replacement of RGC and/or regeneration of the optic nerve. Therefore, literature covering transplantation of cell-derived trabecular meshwork cells will not be further detailed. Different cell types have been used to address the issue of RGC replacement. Even though PSCs are currently considered as the most promising source of cells for cell replacement, some proof of concept and informative studies reporting retinal transplantation feasibility have been performed with other cell types, such as RGC precursors or adult neural stem cells.

### Non-pluripotent Stem Cells

Transplantation of embryonic or young postnatal RGC isolated from embryonic retina of green fluorescent protein (GFP)-mice showed the presence of GFP-positive cells in the retina of axotomized adult rats (Hertz et al., 2014; Venugopalan et al., 2016). Despite a low percentage of successful transplantation (around 10%), some engrafted GFP-positive cells harbored neurite elongation on the retinal surface with diverse dendrite architecture. Interestingly, recording engrafted cells revealed a response to light indicating a functional integration of grafted RGCs into the retinal circuitry (Venugopalan et al., 2016).

Other pioneer works have been performed with adult neural stem cells isolated from the hippocampus and expanded *in vitro*, showing that intravitreal injections of these cells can result in integration at some extend in young neonatal retina (Takahashi et al., 1998; Suzuki, 2003; Van Hoffelen et al., 2003) or dystrophic retina (Young et al., 2000; Mellough et al., 2004) but not in normal adult retina (Young et al., 2000). Interestingly, Mellough et al. (2004) reported the presence of grafted cells 4 and 8 weeks after intravitreal injection in the RGC layer of RGC-depleted mouse retina by previous intraocular administration of *N*-methyl-D-aspartate (NMDA). Even if the authors failed to detect the expression of specific RGC markers such as Brn3b, grafted cells were immunoreactive for a neuronal-specific marker β3-tubulin, suggesting that the *in vivo* neural maturation of grafted cells was possible.

Depending on the species, different endogenous neurogenic sources allow the generation of all retinal cell types in adults and Muller glial cells (MGC) have been of particular interest (Jadhav et al., 2009; Fischer and Bongini, 2010; Ramachandran et al., 2010; Goldman, 2014). Notably, mature adult MGCs have shown to maintain some characteristics of mammalian retinal progenitors (Reichenbach and Bringmann, 2013; Surzenko et al., 2013) after ectopic expression of Ascl1 (Pollak et al., 2013; Ueki et al., 2015) and have the potential to be amplified *in vitro* (Limb et al., 2002; Lawrence et al., 2007). For this reason, MGCs have been considered a good candidate for retinal transplantation and surprisingly, MGCs can efficiently differentiate into RGC-like cells *in vitro* after ectopic expression of key factors favoring RGC fate (Song et al., 2013, 2016) or in presence of FGF2 and a Notch pathway inhibitor (Singhal et al., 2012). Transplantation of these MGC-derived RGC-like cells in NMDA-injured rat retina, revealed a few cells expressing RGC markers like Isl1 in the host RGC layer, 4 weeks after transplantation. A modest functional improvement was observed while grafted cells failed to extend long processes toward the optic disk reflecting a neuroprotective effect. Similar results have been also obtained with RGC-like cells derived from feline MGCs (Becker et al., 2016).

### Pluripotent Stem Cells

Different groups have explored the ability of mouse ESC-derived neural progenitors (NPs) to integrate and differentiate into the retina. Using GFP-expressing ESC-derived NPs, Meyer et al. (2006) reported the presence of GFP-positive cells with RGC-like morphology in the ganglion cell layer (GCL), 16 weeks after intravitreal injection in mouse model of Batten disease, characterized by neuronal lost in many regions of the CNS, including the retina. Similar results have been obtained after intraocular injection of mouse ESC-derived NPs into young post-natal rats without any retinal lesion (Jagatha et al., 2009). The immature state of the host retina may account for the ability for some transplanted cells to integrate into the retina as previously reported with embryonic NPs (Van Hoffelen et al., 2003).

Focusing on RGC degeneration, retinal-like structures derived from GFP-expressing mouse ESCs have been injected in RGC-depleted mouse retina (Aoki et al., 2008). In this condition, the presence of ESC-derived retinal cells expressing some RGC markers (Tuj1 or Brn3a) can be observed in the remaining GCL of NMDA-injured retina. However, the main limitation of these different studies is the weak characterization of the injected cells, as illustrated by teratoma formation in half of the animals (Aoki et al., 2008), a potential risk also reported with injection of mouse ESC-derived retinal progenitors (Cui et al., 2013). In order to address this issue, developing strategies to deplete the transplantable cell population from PSCs is required. One strategy aiming at forcing the differentiation of mouse iPSCs into RGC-like cells has been developed by overexpressing *Atoh7* before transplantation (Chen et al., 2010). In this condition, 2 weeks after transplantation into NMDA-injured adult mouse retina, RGC-like cells were found in the vitreous, close to the retinal surface, but never integrated into the retina. An excessive stage of differentiation of engrafted cells may explain integration failure, highlighting the importance of the differentiation stage for cell therapy. A more recent study using GFP-expressing ESC-derived NPs has assessed the functionality of injected cells in two different models of RGC degeneration (DBA/2J mice and NMDA-injured retina) (Divya et al., 2017). The authors showed a modest integration of grafted cells in both models and a relative improvement of visual function only in NMDA-injected mice. The phenotype of integrated cells was not precisely characterized but according to the authors, c-fos immunoreactivity of the transplanted cells could suggest functional integration in the retinal circuitry. Additionally, no projection into the optic nerve from GFP-positive transplanted cells was observed, suggesting, as also reported by Satarian et al. (2013), that grafted cells could provide a neuroprotective support limiting the cell death induced by NMDA, which could explain the functional benefit observed. The authors hypothesized that integration failure of injected cells in DBA2J mice could be due to an inherent high IOP.

Cell sorting methods based on the expression of specific cell-surface markers allow the enrichment of a cell type of interest. They represent an improvement in order to transplant an homogenous and well-characterized cell population, devoid of PSCs. Parameswaran et al. (2015) performed transplantation of mouse PSC-derived Thy1-positive RGC-like cells after isolation carried out using MACS method and anti-mouse CD90.2 (Thy1.2) magnetic particles. Immunohistochemical analysis 2–4 weeks after transplantation in a rat model of glaucomatous neuropathy with high IOP revealed the presence of few cells expressing two RGC markers, Brn3b and β3-tubulin, in the host retina (Parameswaran et al., 2015).

Altogether, these data collected using mouse ESC or iPSC-derived neural or retinal cells are promising even if their interpretation may be reconsidered to some extent in view of recent data observed after allogenic transplantation of photoreceptors. Indeed, refinement in the follow-up of injected cells has revealed that material transfer or exchange between donor and host cells can be observed rather than real donor cell integration (Pearson et al., 2016; Santos-Ferreira et al., 2016; Singh et al., 2016; Waldron et al., 2018).

Transplantation of human PSC-derived RGCs is currently poorly documented. Banin et al. (2006) have been the first to test the ability of transplanted human ESC derivatives, i.e., NPs, to survive and integrate into the rat retina after injection either in the subretinal space or in to vitreous (Banin et al., 2006). Transplanted cells expressed some key regulator genes of retinal development such as VSX2 and PAX6 and the authors hypothesized that microenvironment would help transplanted cells to differentiate into retinal neurons. Few transplanted cells can be detected in the host GCL but their precise phenotype was not explored further. Similar observation has been reported by Lamba et al. (2009), after intravitreally injection of uncharacterized human ESC-derived retinal cells in neonatal mice (Lamba et al., 2009). Since their study was mainly dedicated to the integration of photoreceptor-like cells in a model of outer retina impairment, the phenotype of cells integrated into the inner retina was not more detailed.

An alternative approach using engineering biomaterials has been recently reported, where human iPSC-derived RGCs were seeded on a biodegradable poly lactic-co-glycolic acid (PLGA) scaffold. According to morphological and functional criteria, *in vitro* analysis revealed nice differentiation and maturation of iPSC-derived RGCs after seeding on PLGA scaffold. Implantation of the engineered human RGC-scaffold biomaterial by posterior sclerotomy in both rabbit and monkey retina did not revealed any sign of rejection; unfortunately, the phenotype of survival cells has not been explored further (Li et al., 2017). Since attachment of grafted cells precisely onto the surface of the host retina is critical, the use of an RGC-scaffold biomaterial should limit the diffusion of engrafted cells in the vitreous.

Despite the high potential of human PSCs for cell therapy application, some obstacles linked to intrinsic properties of PSCs represent some impediment to the development of cell therapy. Because these cells have the faculty to generate tumors, all efforts have to be done to eliminate any remaining PSCs from the transplantable cell population (Conesa et al., 2012; Lee et al., 2013). Methodological improvement and cell-sorting methods allowing the generation and isolation of well-characterized cells of interest will also improve the safety.

## RGC Disorders: Cell Therapy and the Challenge of Optic Nerve Regeneration

Significant progress has been performed to provide cell sources suitable for transplantation approaches. However, all these efforts would be useless if in parallel any progress could be done concerning the ability of neurons, e.g., RGCs, to regenerate their axon in adult, allowing rewiring of visual pathways. Important progress has also been realized regarding this issue and extensively reported in different reviews (Fischer and Leibinger, 2012; Crair and Mason, 2016; Leibinger et al., 2016; Benowitz et al., 2017; Laha et al., 2017).

### Endogenous Regeneration Capacity

Glial cells have multiple role during degenerative and regenerative process. Oligodendrocytes, the myelinated cells of the CNS express different inhibitory factors for axon regrowth (Geoffroy and Zheng, 2014) and removing myelin-associated proteins such as NOGO enhances optic nerve regeneration (Fischer et al., 2004; Schwab, 2004). Injury-induced glial scar or more generally gliosis constitute also a hindrance to axon outgrowth (Koprivica et al., 2005; Qu et al., 2010; Usher et al., 2010; Nickells et al., 2012), even though some evidence of positive effect of gliosis on neuronal survival and regenerative process have been reported (Leibinger et al., 2009; Benowitz et al., 2017; Laha et al., 2017). Different intrinsic modifications after developmental phase contribute also to axon outgrowth limitation (Goldberg et al., 2002). For example, deletion of cytokine signaling 3 (SOCS3), transcription factor KLF4 or Phosphatase and tensin homolog (PTEN), a negative regulator of the mammalian target or rapamycin (mTOR) have been shown to strongly stimulate axon regeneration after optic nerve injury (Park et al., 2008; Smith et al., 2009; Sun et al., 2011). Interestingly, axon outgrowth was virtually absent beyond the optic chiasm after optic nerve injury.

Manipulation of the environment can also promote axon regeneration, as demonstrated for example by the co-administration of Osteopontin and IGF-1 for a specific subtype of RGCs (Duan et al., 2015). Oncomodulin, a macrophage-secreted factor can also promote axon regeneration (Yin et al., 2006, 2009) and combined with a cAMP analog and PTEN deletion, de Lima et al. (2012) have been able to demonstrate a full-length axon regeneration after optic nerve injury associated with a transitory visual function recovery. Finally, we can cited exogenous electrical activity as an important process for RGC survival and regeneration (Goldberg et al., 2002; Morimoto, 2012; Lim et al., 2016). Interestingly, many of these processes are complementary, as recently demonstrated by the combination of enhancement of electric activity with PTEN/SOCS3 deletion or osteopontin, IGF-1 and CNTF delivery via Adeno-associated virus injection, that led to an important RGC regeneration and visual function enhancement (Bei et al., 2016).

Recent progress that make possible long-length axon regeneration is considerably promising but leads also to new challenges. Rewiring visual pathways will enable some restoration of visual function only if axons terminals reach their target respecting the topographical arrangement of visual projections (Lemke and Reber, 2005; Huberman et al., 2008; Assali et al., 2014; Lim et al., 2016; Kuwajima et al., 2017; Ito and Feldheim, 2018). In the same way, transplanted RGCs have to connect retinal partners, i.e., bipolar and amacrine cells with regard to precise partners (Seung and Sümbül, 2014; Demb and Singer, 2015; Mauss et al., 2017) in order to send a pertinent signal to high-order visual structures.

### Cell Transplantation – Axon Regeneration Support

Because cell replacement for RGC disorders can appear unrealistic short term, some groups aim at developing cell-based therapies suitable for neuroprotective and regenerative support to endogenous RGCs (Johnson et al., 2011). Different cell sources have been tested for this purpose (Mead et al., 2015) but MSCs are probably one of the best candidates. MSCs are unable to differentiate into retinal cells and to integrate into the retina (Hill et al., 2009) but have been reported to operate paracrine activity supporting RGC neuroprotection in some models of RGC injury (Yu et al., 2006; Johnson et al., 2010; Levkovitch-Verbin et al., 2010; Harper et al., 2011; Paul and Anisimov, 2013; Teixeira et al., 2013; Mesentier-Louro et al., 2014; Mead et al., 2015, 2016; Ding et al., 2017). This neuroprotective effect has been attributed alternatively to Platelet-derived growth factor, NGF, BDNF, or Neurotrophin-3 secretion (Johnson et al., 2011, 2014; Mead et al., 2013; Osborne et al., 2018). Interestingly, this trophic support was reported to affect both RGC survival and axon regeneration. Microvesicles released by MSCs could also mediate in part the neuroprotective effect of these cells (Yu et al., 2014; Yang T.C. et al., 2017), by the delivery of specific neutrophic factor and/or miRNAs (Mead and Tomarev, 2017; Mead et al., 2018). Finally, in agreement with the role of inflammation in optic nerve regeneration (Yin et al., 2006, 2009; Hauk et al., 2010), modulation of immune and inflammatory responses has been partially attributed to MSCs (Lee et al., 2015; Mac Nair and Nickells, 2015).

iPSC-derived NPs are also believed to deliver neuroprotective support (Satarian et al., 2013). Functional and histological analysis showed some protection due to intraocular injection of iPSC-derived NPs, few days after optic nerve crush (Satarian et al., 2013). Histological analysis revealed the presence of engrafted cells in the GCL immunoreactive for pan-neuronal markers (Neurofilament, microtubule-associated protein 2) but without long distance neurite outgrowth. This observation led the authors to attribute the functional benefit to the secretion of neurotrophic factors instead of cell integration, strengthened by *in vitro* release of CNTF, FGF2, and IGF-1 by the iPSC-derived NPs (Satarian et al., 2013).

## Perspectives and Future Directions

Much progress has been made toward differentiating human PSCs into RGCs thanks to our knowledge of *in vivo* retinal development. Even though the majority of the RGC differentiation protocols described above use animal-derived products some of these protocols have been adapted to a completely defined xeno-free system (Sridhar et al., 2013; Lee et al., 2016; Reichman et al., 2017) for the generation of GMP-compliant RGCs compatible for transplantation. Another key issue for a future stem cell-based therapy is the purification of donor cell type with elimination of contaminating cells; a strategy that has been started by targeting the cell surface antigen THY1 to isolate an homogenous population of RGCs (Gill et al., 2016). With protocols developed to generate highly enriched populations of human RGCs, further *in vivo* transplantation studies are required to understand how grafted RGCs can integrate into the GCL. RGC transplantation pose other significant challenges: (i) connecting RGC dendrites with presynaptic amacrine and bipolar cells in host retina; (ii) extending RGC axons radially to the optic nerve head and (iii) targeting the central nervous system through the optic nerve tract. Attachment of grafted cells precisely onto the surface of host retina is therefore critical. The absence of physical support for injected cells is also a possible limitation for their survival and correct axon regeneration, as recently demonstrated with the use of biomaterial or synthetic polymers to orientate RGC axon growth *in vitro* (Kador et al., 2014; Sluch et al., 2015; Li et al., 2017; Yang Y. et al., 2017). Introducing a RGC-scaffold biomaterial into the eye is technically challenging, compared to simple injection of cells into the vitreous cavity. Nevertheless, successful transplantation of engineered RGC-scaffold biomaterial has been reported recently in monkey eyes (Li et al., 2017), paving the way of the generation and transplantation of complex 3D bio-scaffolds. In this context, combining the use of specific scaffolds and 3D bioprinting has the potential to control RGC positioning. Recent papers, demonstrated that RGCs can be successfully printed without loss of viability and some phenotypic features, such as neurite outgrowth and electrophysiological responses (Lorber et al., 2014; Kador et al., 2016). Further advances in bioprinting research, particularly with the use of neurons that cannot be easily manipulated by printing, should facilitated the development of novel cell therapies aiming at promoting neural regeneration, that could be used for RGC replacement in different ocular diseases.

Lastly, the rejection of grafted cells inherent to transplantation approaches remains an important challenge for cell therapy targeting RGCs. Producing specific patient iPSCs and autologous transplantation should bypass the problem of immune rejection but this customized cell therapy is extremely expensive and time-consuming. Alternatively, the development of a bank of iPSC lines designed to match the human leukocyte antigen (HLA) cell type should limit immune cell response and reduce the financial cost.

Whilst many challenges remain, the exciting progress made in these pioneering studies offer a hope for patients with untreatable advanced-stages RGC disorders. Improvement of RGC production from PSCs and regenerative technologies offer the opportunity to consider the replacement of lost cells and visual restoration, not only stabilization of the remaining visual acuity.

## Author Contributions

All authors participated in the conception of the review and literature search. GO wrote the first draft of the manuscript. OR and OG wrote sections of the manuscript and prepared the tables. OR and GO prepared the figures. All authors approved the final version.

## Conflict of Interest Statement

The authors declare that the research was conducted in the absence of any commercial or financial relationships that could be construed as a potential conflict of interest.
